# Chronological analysis of periodontal bone loss in experimental periodontitis in mice

**DOI:** 10.1002/cre2.806

**Published:** 2023-11-23

**Authors:** Cristhiam de J. Hernández Martínez, Pedro Felix Silva, Sergio L. Salvador, Michel Messora, Daniela B. Palioto

**Affiliations:** ^1^ Department of Oral & Maxillofacial Surgery and Periodontology, Ribeirão Preto Dental School University of Sao Paulo—USP Ribeirão Preto SP Brazil; ^2^ Department of Clinical Analyses, School of Pharmaceutical Sciences of Ribeirao Preto University of Sao Paulo—USP Ribeirão Preto SP Brazil

**Keywords:** alveolar bone loss, host microbiota interactions, inflammation, periodontal diseases

## Abstract

**Objectives:**

Periodontal disease is understood to be a result of dysbiotic interactions between the host and the biofilm, causing a unique reaction for each individual, which in turn characterizes their susceptibility. The objective of this study was to chronologically evaluate periodontal tissue destruction induced by systemic bacterial challenge in known susceptible (BALB/c) and resistant (C57BL/6) mouse lineages.

**Material and Methods:**

Animals, 6–8 weeks old, were allocated into three experimental groups: Negative control (C), Gavage with sterile carboxymethyl cellulose 2%—without bacteria (Sham), and Gavage with carboxymethyl cellulose 2% + *Porphyromonas gingivalis* (Pg‐W83). Before infection, all animals received antibiotic treatment (sulfamethoxazole/trimethoprim, 400/80 mg/5 mL) for 7 days, followed by 3 days of rest. Microbial challenge was performed 3 times per week for 1, 2, or 3 weeks. After that, the animals were kept until the completion of 42 days of experiments, when they were euthanized. The alveolar bone microarchitecture was assessed by computed microtomography.

**Results:**

Both C57BL/6 and BALB/c mice exhibited significant bone volume loss and lower trabecular thickness as well as greater bone porosity compared to the (C) and (Sham) groups after 1 week of microbial challenge (*p* < .001). When comparing only the gavage groups regarding disease implantation, time and lineage, it was possible to observe that within 1 week of induction the disease was more established in BALB/c than in C57BL/6 (*p* < .05).

**Conclusions:**

Our results reflected that after 1 week of microbial challenge, there was evidence of alveolar bone loss for both lineages, with the loss observed in BALB/c mice being more pronounced.

## WHY THIS PAPER IS IMPORTANT

- After 1 week, there was evidence of alveolar bone loss for both lineages.
- The bone loss observed in the BALB/c lineage was more pronounced.
- 2 weeks were necessary to consolidate the process and make it chronic.
- 3 weeks did not seem to influence bone loss at the end of the analyzed period.

## 1 INTRODUCTION

Over time, the understanding of periodontitis has transitioned from a simple infectious disease paradigm to a more complex dysbiotic condition, marked by the disruption of microbial communities (Hajishengallis & Lamont, 2021). This dysbiosis disrupts the delicate balance of tissue homeostasis, leading to an aberrant immuno‐inflammatory response and subsequent tissue damage (Hajishengallis & Moutsopoulos, 2015). The activation of receptors such as Toll‐like receptors (TLRs) triggers the production of inflammatory cytokines and chemokines, culminating in pathological bone loss and eventual destruction of the dental supporting structures (Li & Amar, 2007; Polak et al., 2009; Shimabukuro et al., 2021; Wu et al., 2020). Osteoclasts play a pivotal role in this intricate process, as increased osteoclast formation and activity contribute to the host's secretion of proinflammatory cytokines (Lin et al., 2021; Palioto et al., 2019; Wu et al., 2020). The variability in this orchestrated process is intimately linked to the host's susceptibility or hyperresponsive profile, which, in turn, influences the transition from a commensal biofilm state to a pathogenic one and the development of chronic resorptive disease.

One noteworthy protagonist in the realm of periodontal disease is *Porphyromonas gingivalis*, a key microorganism recognized for its significant role in disease development (Ding et al., 2018). *P. gingivalis*, in conjunction with other microorganisms, substantially contributes to the onset of severe periodontitis and its associated systemic consequences (Graves et al., 2012). This microorganism incites an uncontrolled and destructive immune response within the context of disrupted homeostasis (Gully et al., 2014; Nagashima et al., 2017). Host‐related factors, including genetic and epigenetic components, alterations in signaling pathways, rare immune deficiencies, and environmental influences, are pivotal in determining the susceptibility to immune subversion by *P. gingivalis* and the induction of dysbiosis (Hajishengallis & Lamont, 2021). Interestingly, certain individuals may possess intrinsic mechanisms that resist *P. gingivalis's* capacity to transform a eubiotic microbiota into a dysbiotic one, providing insights into the complexity of host–pathogen interactions (Hajishengallis & Moutsopoulos, 2015). The investigation into the chronology of bone loss in a periodontitis model induced in two distinct mouse strains, characterized by differing susceptibilities, unveils the intricate interplay of these multifaceted factors in the pathogenesis of periodontal disease.

The first animal model used to induce alveolar bone loss was generated at the Forsyth Institute in the United States (Sharawy et al., 1966). In the oral gavage model of periodontitis, *P. gingivalis* (Graves et al., 2012) does not cause alveolar bone loss in germ‐free mice, although it can colonize them in biofilm conditions (Baker, Dixon, & Roopenian, 2000). In conventional mice, however, this oral bacterium remodels the periodontal commensal microbiota into a dysbiotic community that causes bone loss, provided that the mice have intact complement and TLR signaling pathways (Baker, 2005; Graves et al., 2012; Hajishengallis & Lamont, 2021; Marchesan et al., 2018; Wilensky et al., 2009). The generated inflammatory impact on the periodontium differs in the levels of gingival epithelial growth (Li & Amar, 2007) in the bone loss profile (Li & Amar, 2007; Polak et al., 2009) and the levels of inflammatory markers (Okada et al., 2010).

Alveolar bone resorption in mice is generally assessed around the upper molars, as the induction of bone loss in the lower molars is slower due to thicker cortical alveolar bone and wider buccolingual dimensions (Wilensky et al., 2009). Bone loss can be measured histologically by macroscopic analysis or microcomputed tomography. The latter, however, is able to observe the bone microstructure, trabeculae, and porosity and offers a better way of observing this complex structure's tridimensionality (Li & Amar, 2007; Wu et al., 2020).

Much research has been designed to study methods for initiating experimental periodontal lesions with *P. gingivalis*. However, all these methods lead to variations in the time for periodontal lesions to develop, ranging from 10 days (Li & Amar, 2007) to 6 weeks after final inoculation (Baker, 2005), and recent studies have shown that bone loss can be detected 2 weeks after gavage (Palioto et al., 2019) or 3 weeks after the onset of infection (Marchesan et al., 2018).

Therefore, we used this model of experimental periodontitis induced by systemic microbial challenge (gavage) to assess the dynamic microstructural changes in alveolar bone. It is, therefore, essential to better characterize this model to allow reproducibility in experimental investigations, making it relevant to human pathology. To the best of our knowledge, no study has chronologically evaluated periodontal tissue degradation after experimental periodontitis induced by *P. gingivalis* in two lineages of C57Bl/6 and BALB/c mice, allowing comparison by week of induction and susceptibly different mouse lineages.

## 2 MATERIALS AND METHODS

### 2.1 Ethical considerations

The experimental protocols in this study were approved by the Ethics Committee on Animal Use (CEUA) of the Ribeirão Preto School of Dentistry (FORP) of the University of São Paulo (USP) with protocol number CEUA 2018.1.644.58.6. Every effort was made to minimize the suffering of the animals, as well as the number of animals used, in accordance with the guidelines of the Brazilian Society for Laboratory Animal Science (SBCAL/COBEA), respecting the ethical principles of animal experimentation, the standards for the didactic‐scientific practice of vivisection of animals (Law 11.794/2008), the Universal Declaration of Animal Rights of UNESCO (United Nations Educational, Scientific and Cultural Organization) and international standards (Guide for the Care and Use of Laboratory Animals, National Academy of Science, USA, 1996).

### 2.2 Calculation of sample size

The sample size calculation was performed using GraphPad Statemate 2.0 software (GraphPad Software). The optimal sample size to ensure 80% power in the statistical analysis of the data obtained in this study was calculated by considering the differences in means and standard deviations between the DP and C groups of the study by Oliveira et al. (2017), recognizing the significant difference of 5% (*δ*) between the groups, 95% confidence interval (*α* = .05), standard deviation (*σ*) of 23%, and the changes in the bone volume (VO) mean as the primary variable [Z*α* (1.96) + Z*ß* (0.84)]2 = 7.84. The calculation of the sample per group was based on the formula: *n* ≥ {2[(*σ*)2/(*δ*)2]} × (Z*α* + Z*ß*)2. The minimum number required was eight animals per experimental group. Considering possible losses related to the sensitivity of the animals to the experimental periodontitis induction protocols, 10 mice were included per subgroup for each experimental group (BALB/c & C57BL/6).

### 2.3 Study design

We used 140 mice (*Mus musculus*), 70 BALB/c and 70 C57BL/6. To avoid the possible effects of estrogen, we used only 6‐week‐old male mice weighing approximately 20 g from the PUSP‐RP Central Animal facility. The mice were housed in collective autoclavable polypropylene cages under a temperature ranging from 22°C to 24°C with 12/12‐h light–dark cycles. The animals underwent a period of 7 days of acclimatization to the environment and the project's execution team. During the study, the animals were fed a selected solid ration and water ad libitum.

**Figure 1:**
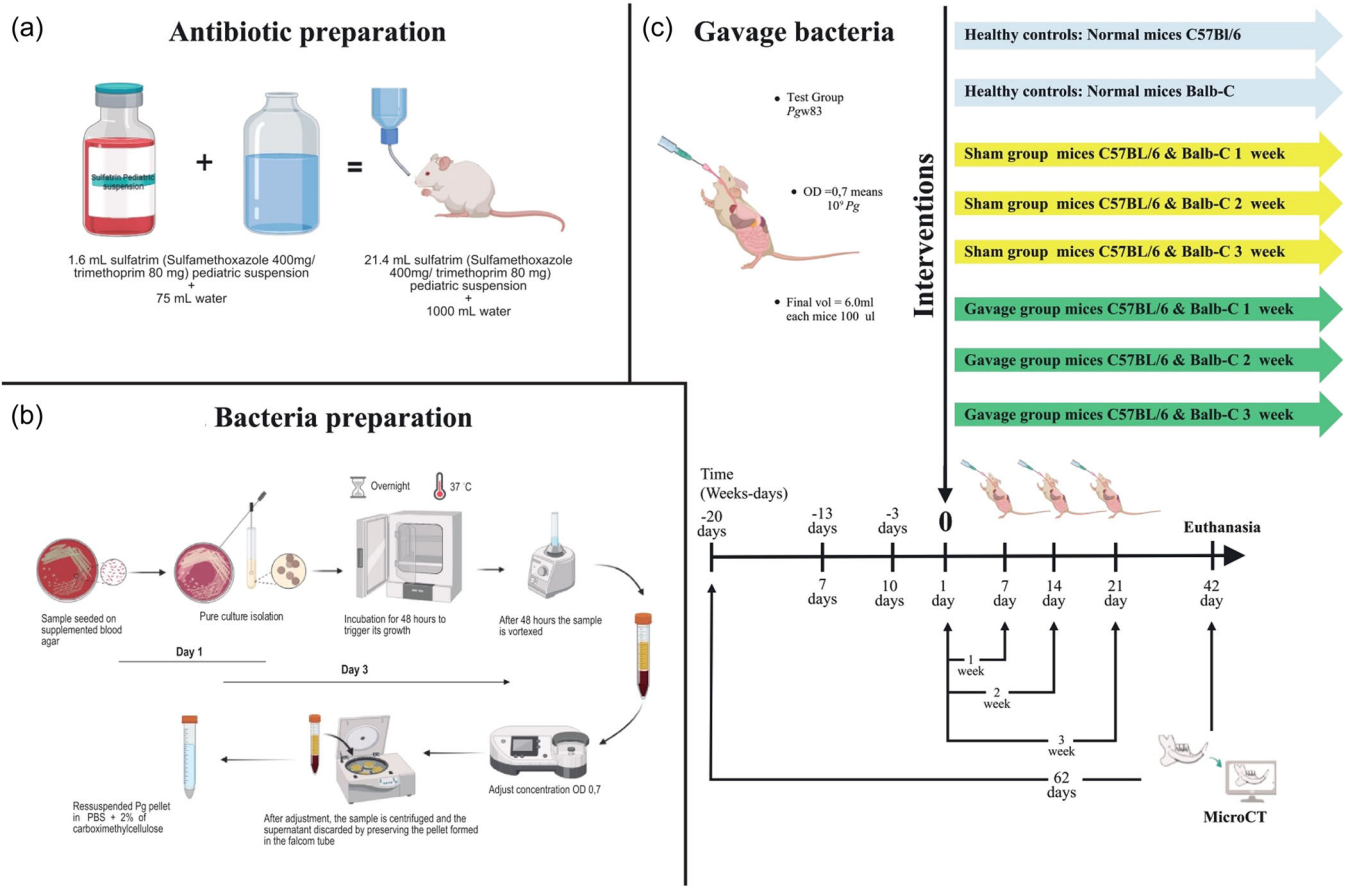
Experimental design. (a) Antibiotic preparation. (b) Bacteria preparation. (c) Gavage bacteria.

The animals were identified by a numerical code and allocated into three different subgroups: Control (C), Sham Gavage with 2% sterile carboxymethyl cellulose (Sham), and *Pg* W83 (Gav). Mice received the antibiotic trimethoprim‐sulfamethoxazole systemically at a concentration of 80 mg trimethoprim and 400 mg sulfamethoxazole in 5 mL deionized water ad libitum for 10 days to reduce the native flora, followed by a 3‐day period without antibiotics (Figure 1a).

### 2.4 Preparation of the *P. gingivalis* bacterial culture

The original *Pg* W83 culture was kept frozen in defibrinated sheep blood at −80°C, and an aliquot of the bacteria was maintained by weekly transfer on supplemented blood agar (trypticase soy agar with 0.1% yeast extract, 5.0 μg of hemin per mL, 0.5 μg of menadione per mL and 5% defibrinated sheep blood). For the experiments, the bacteria were grown in anaerobiosis under an atmosphere of 5% CO_2_, 10% H_2_, and 85% N_2_ on supplemented blood agar at 37°C for 4–7 days. The bacteria were suspended in phosphate‐buffered saline (PBS), and the number of colony‐forming units (CFU) was standardized using the optical density at 700 nm (Baker & Roopenian, 2002; Baker et al., 1999; Hart et al., 2004) (Figure 1b).

### 2.5 Systemic microbial challenge

The experimental group was inoculated by gavage with a total of 10^9^ live *P. gingivalis* CFU in 200 µL of PBS and 2% sterile carboxymethyl cellulose through a feeding needle. The total volume was placed in the stomach, leaving extravasation in the oral cavity (Kato et al., 2018). This suspension was administered three times at intervals of 2 days per week until completion of 3 weeks, and the experiment was divided into four repetitions per subgroup and strain to minimize and control possible errors (Palioto et al., 2019) (Figure 1c).

The animals were euthanized 62 days after the beginning of the experiments. Euthanasia was performed by intraperitoneal administration of a lethal dose of pentobarbital (150 mg/kg).

### 2.6 Analysis with X‐ray transmission computed microtomography (micro‐CT)

**Figure 2:**
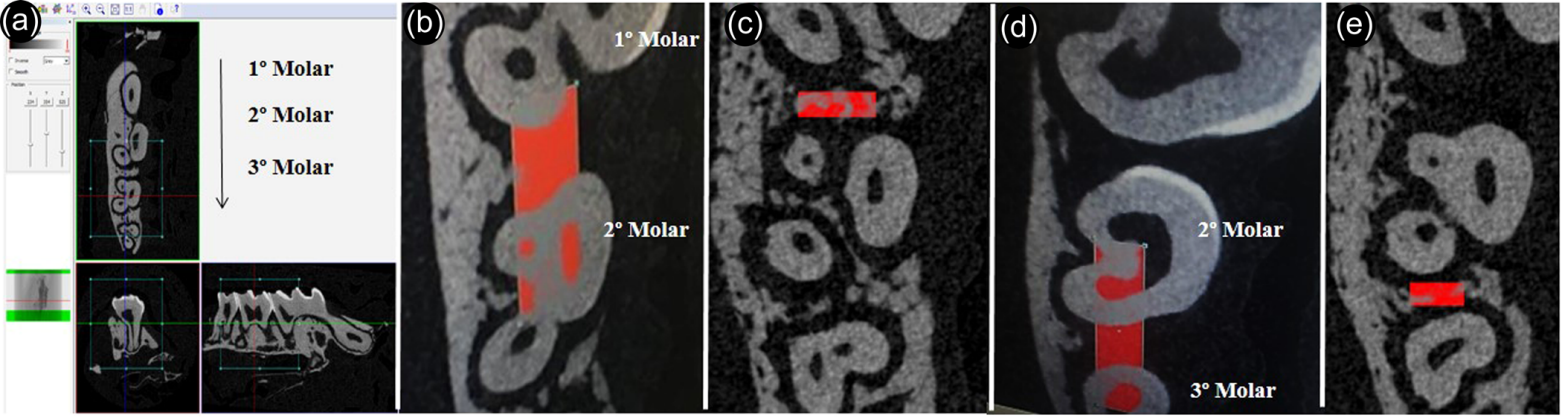
Micro‐CT analysis. (a) Measurement in the CT Analyzer software in the interproximal region for volumetric analysis by means of a sagittal image of the maxillary second molar. (b) Delimitation of the mesial region of interest (ROI) mesial interproximal region. (c) Measurement of mesial region 4. Delimitation of the ROI distal interproximal region. (d) Measurement of the distal region.

Nondemineralized specimens were scanned using a cone beam micro‐CT system (Skyscan 1174; Bruker). The X‐ray generator was operated at an acceleration potential of 60 kV with a beam current of 165 µA and an exposure time of 490 ms per projection. Images were produced with a voxel size of 6 × 6 × 6 µm. Using appropriate software (Data Viewer, version 1.5.0; Bruker), the generated three‐dimensional models were rotated to a standard analysis position (Figure 2a).

Volumetric analysis of the upper second molar mesial and distal interproximal regions was performed (Shimabukuro et al., 2021). After identification of the cementoenamel junction, 15 sections were counted apically. Section 16—the region of interest—was delimited until reaching another 15 sections for analysis. Briefly, for the mesial interproximal measurement, a rectangle was created from the palatal pulp chamber of the maxillary second molar, extending to the buccal‐distal pulp chamber of the maxillary first molar, thus delimiting the width (Figure 2b). After defining the width, the rectangle was reduced in length to the interproximal region without changing its width to standardize and cover only the alveolar bone (Figure 2c). For the analysis of the distal interproximal region, a rectangle was created from the distal pulp chamber of the maxillary second molar and the mesial pulp chamber of the maxillary third molar (Figure 2d) standardized and covered only the distal interproximal alveolar bone (Figure 2e). All micro‐CT analyses were performed by a calibrated operator who was unaware of the experimental groups and treatments performed.

Linear measurements of the alveolar bone level were taken at two different sites, mesial and distal (interproximal), as previously described (Messora et al., 2016) on the maxillary second molar. The two linear measurements obtained from each animal were added together to express the value of the alveolar bone level.

### 2.7 Statistical analysis

Two‐way analysis of variance followed by Tukey's test was used to compare the bone parameters obtained from the control and treatment groups. The comparison between each treatment as a function of time was performed. A *p* < .05 was used as the minimum statistical significance value. GraphPad Prism 9.0 software was used to prepare graphs and perform analyses.

## 3 RESULTS

**Figure 3:**
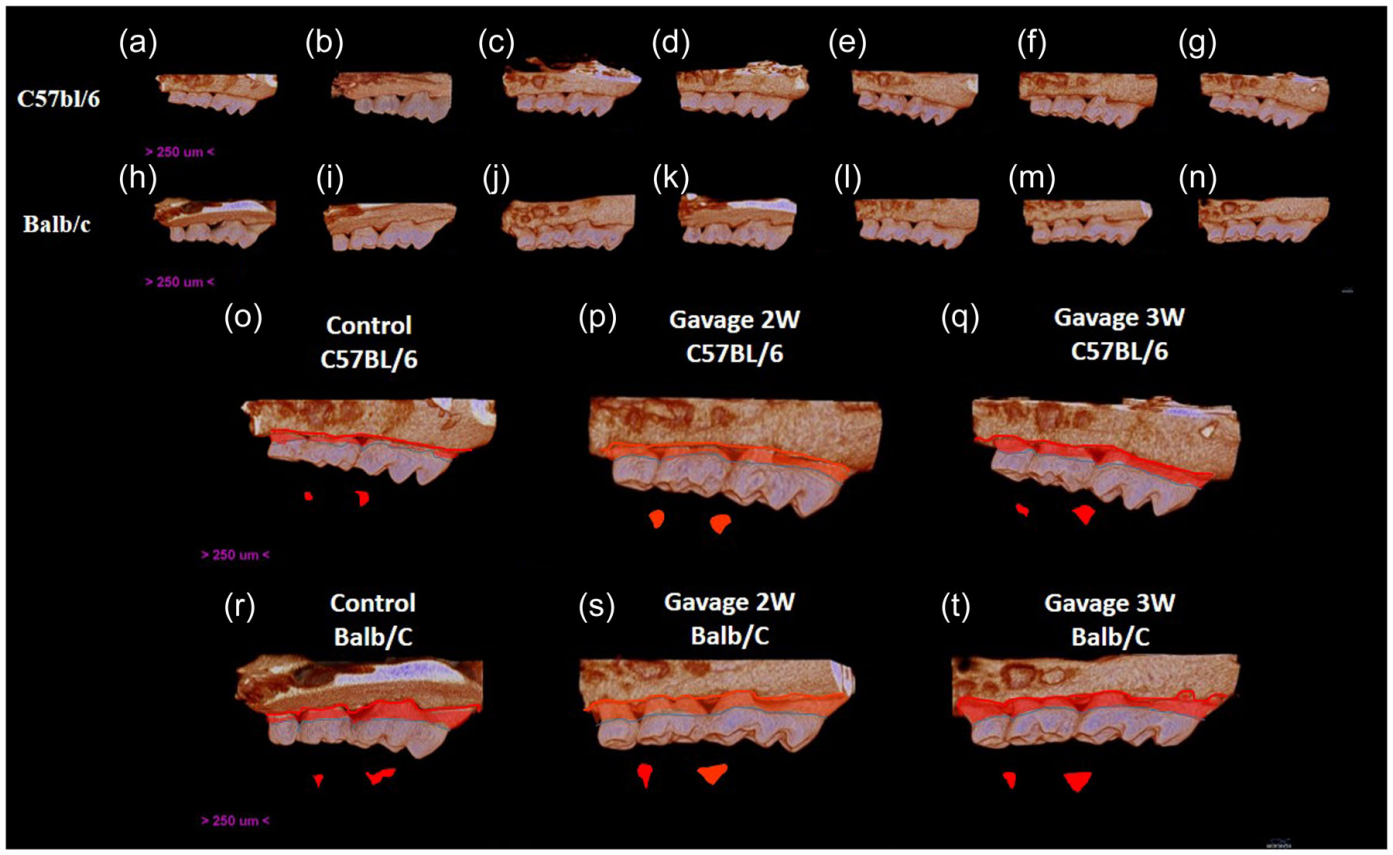
Three‐dimensional rendered reconstructions of the microtomographic sections buccal view of Groups C (a and h), Sham 1 week (b and i), 2 weeks (c and j) and 3 weeks (d and k), Gav 1 week (e and l), 2 weeks (f and m), and 3 weeks (g and n) and representation of the chronicity of the disease in Groups C (o and r) and Gav 2 weeks (p and f) and 3 weeks (q and t); the red drawings represent the size of the area without bone.

Representative images of the three‐dimensional rendered reconstructions of the microtomographic sections of the hemimandibles of the animals in the three groups can be observed in Figure 3.

**Figure 4:**
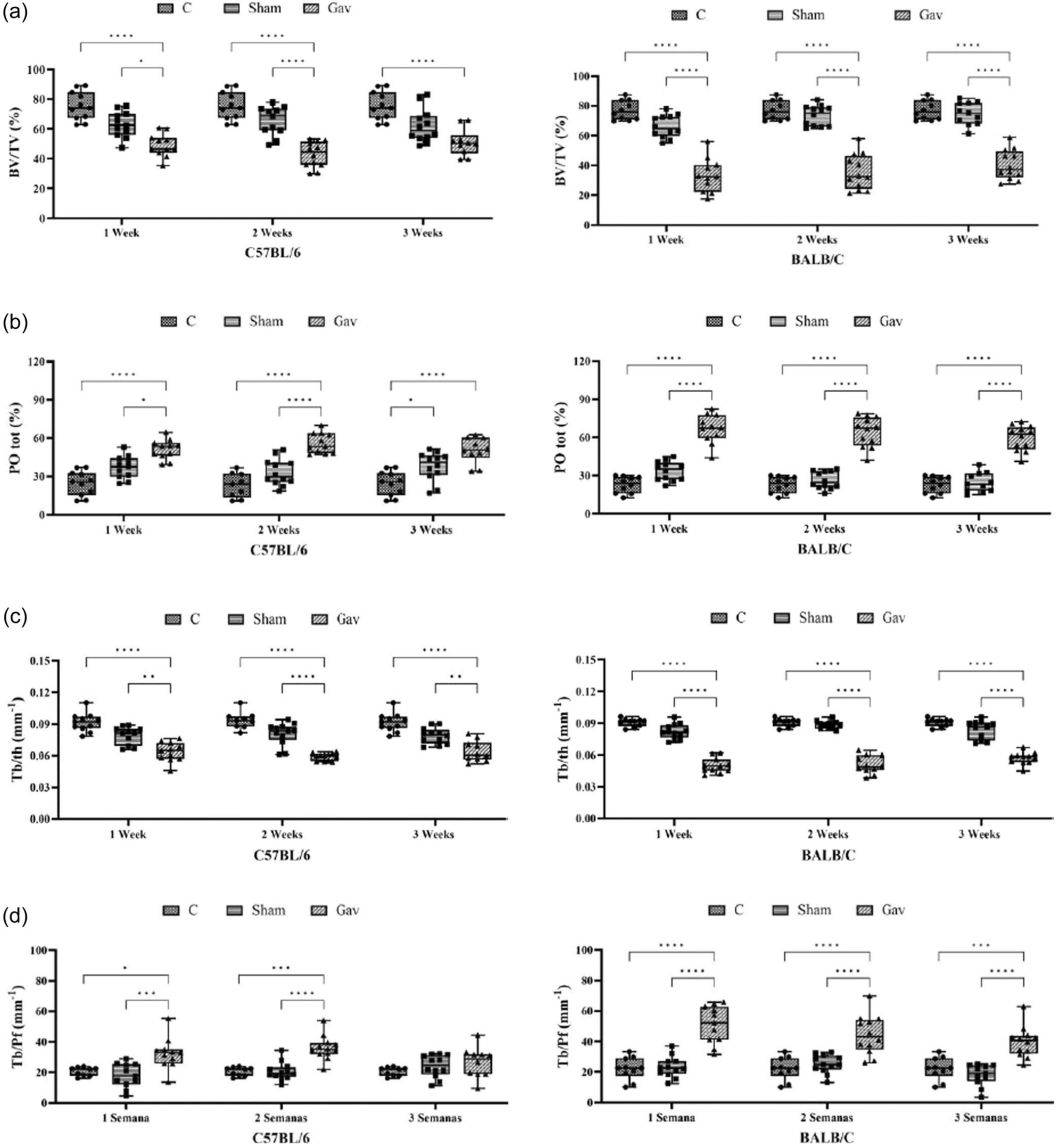
Means and standard deviations of bone volume Bv/Tv (a), total porosity POtot (b), thickness Tb.Th (c), and trabecular pattern Tb.Pf (d) and comparisons between groups. Asterisks indicate significant differences between groups (analysis of variance, Tukey, *p* < .05).

**Figure 5:**
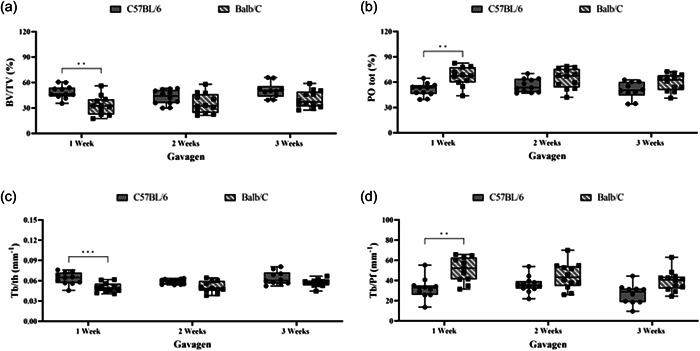
Means and standard deviations in the gavage group. Bone volume Bv/Tv (a), total porosity POtot (b), thickness Tb.Th (c), and trabecular pattern Tb.Pf (d) and comparisons as a function of time, lineage, and implantation of periodontal disease in the gavage group. Asterisks indicate significant differences between groups (analysis of variance, Tukey, *p* < .05).

The means and standard deviation of (Bv/Tv), (PO) tot, (Tb.Th) and (Tb.Pf) are shown in Figure 4. The values showed significantly lower bone volume in the first‐, second‐ and third‐week Gav groups than in the C and Sham groups (*p* < .0001) (Figure 4a). When comparing the bone volume in the Gav group between the two lineages, it is possible to observe that alveolar bone loss is more evident in the BALB/c first‐week (33.29% ± 3.40) (*p* < .01) and second‐week (35.59% ± 3.40) groups when compared to the C57BL/6 first‐week (48.89% ± 2.31) and second‐week (42.78% ± 2.47) groups (Figure 5a).

Figure 4b shows the means and standard deviation of the percentage of total porosity. The results showed significantly increased porosity for the Gav group in both lineages and at all times of induction, followed by the Sham and C groups (*p* < .0001). The BALB/c lineage ‐ Gav group presented the highest percentage of porosity in the first week (66.71% ± 3.40) (*p* < .01) (Figure 5b).

The bone trabecular thickness in mm^2^ is shown in Figure 4c. The first‐, second‐ and third‐week Gav groups had the lowest values for both lineages, which were statistically significant in relation to the other evaluated groups, C and Sham (*p* < .0001). When evaluating only the Gav group for the two lineages, it was possible to observe that the BALB/c first‐week group had a significantly lower value (0.06345 ± 0.002728) than the C57BL/6 first‐week group (0.04 ± 0.002) (*p* < .01) (Figure 5c).

The trabeculae pattern was considerably less organized for the GAV group, within one (30.16 ± 2.324) and 2 weeks (35.69 ± 1.374) of induction when compared to the C and Sham groups in the C57BL/6 lineage (*p* < .001) (Figure 4d). For the BALB/c lineage, a less organized pattern of trabeculae was observed at all times (*p* < .0001) (Figure 4d). When comparing only the gavage groups (Figure 5d), the BALB/c lineage showed the least organized trabecular pattern when compared to the C57BL/6 lineage (*p* < .01).

**Figure 6:**
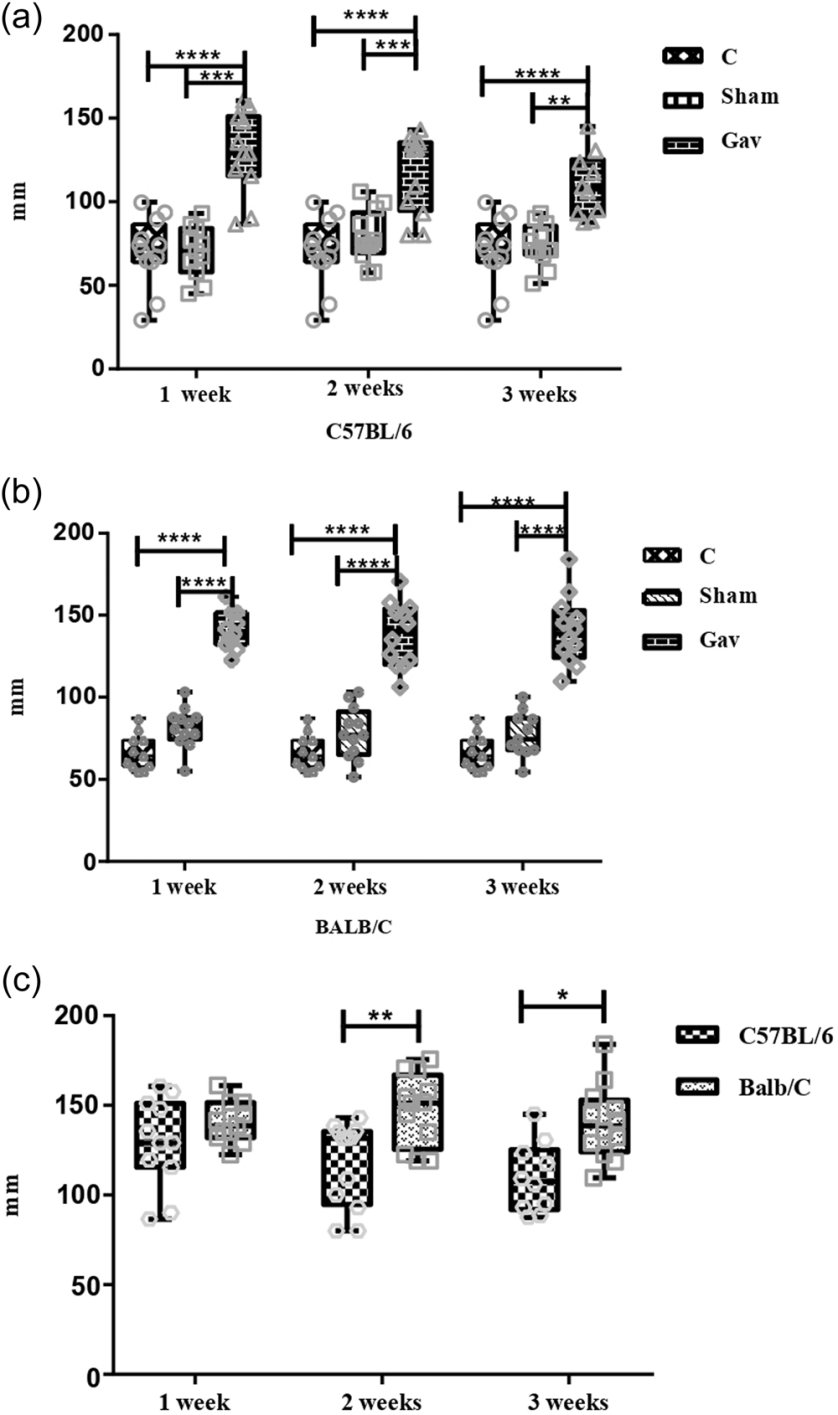
Means and standard deviations of linear measurements of the alveolar bone level. C57BL/6 group (a), BALB/c (b), Gavage groups (c). Asterisks indicate significant differences between groups (analysis of variance, Tukey, *p* < .05).

The gavage groups of both strains showed greater linear bone loss when compared to the Sham and Control groups (*p* < .05) (Figure 6a,b). When comparing only the gavage groups of the two strains, bone loss was evident after 2 weeks of induction in the BALB/c group (Figure 6c).

## 4 DISCUSSION

Knowledge of periodontitis etiopathogenesis has been revised in recent years, and systemic immune‐inflammatory host modulation of the onset and progression of alveolar bone loss is becoming evident. Indeed, the susceptibility of the host to periodontitis and to other chronic diseases might involve a common hyperresponsive inflammatory background that can be expressed or not. Models of experimental periodontitis are often used to understand the process of the disease. In our interpretation, the method of periodontitis induction that would more closely assemble chronic inflammatory periodontitis is, to date, the systemic microbial challenge imposed by a gavage of *P. gingivalis* or the association of other periodontal diseases; the use of a key pathogen appears to be an interesting approach for preclinical studies of periodontitis induction (Abe & Hajishengallis, 2013; Hajishengallis & Diaz, 2020). In this study, we sought to observe the events in bone microarchitecture in two lineages of mice—one known as “susceptible” to the development of periodontitis by means of systemic microbial induction, BALB/c, and another considered “resistant” to it, C57BL/6 (Graves et al., 2012). Additionally, the time of challenge was stratified into the first, second‐, and third weeks of induction. In this way, it was possible to observe the chronology of the process itself.

The gavage model, as demonstrated in previous research (Hajishengallis & Lamont, 2012), has proven invaluable for studying periodontal disease pathogenesis. Faithfully replicating key human disease aspects, including alveolar bone resorption, inflammatory responses, microbial dysbiosis, and systemic effects (Hajishengallis & Lamont, 2016; Hajishengallis et al., 2011; Lamont & Hajishengallis, 2015; Olsen et al., 2016), the gavage model provides a controlled and reproducible means to investigate periodontal disease mechanisms and explore potential therapeutic interventions. While acknowledging that no animal model can fully replicate human disease complexity, the gavage model remains a valuable tool for preclinical studies in periodontitis research.

As has already been reported in the literature, different lineages express distinct susceptibilities to periodontitis induction. However, the results of this study brought about a slightly different concept: the time of induction seems to be different when comparing BALB/c and C57BL/6 mice. BALB/c mice exhibited faster bone loss than C57BL/6 mice. Changes were noted in microarchitecture that demonstrated a worsening in bone quality, with the beginning of trabecular bone thickness and less organized trabecular pattern in the gavage groups from the first week, in addition to high levels of porosity. It is well documented that the ligature‐induced periodontitis model can be started at a known time with a predictable sequence of events that culminates in periodontal bone loss in a few days, which allows it to be a valid and reproducible technique (Baker, Dixon, Evans, et al., 2000; Graves et al., 2012). This standardization is not well established for the gavage‐induced periodontitis model. In many studies, mice are euthanized at 6 weeks after final inoculation (Takeuchi et al., 2013; Yu et al., 2007). Others have shown that bone loss can be detected as early as 2, 3, or even 1 week after initiation of infection (Graves et al., 2012). There is no scientific consensus that demonstrates when the onset, acute and chronic phases of the disease occur.

Studies using the mouse oral gavage model of periodontitis have confirmed that *P. gingivalis* has the ability to inhibit the expression of neutrophil‐recruiting chemokines (Baker, Dixon, & Roopenian, 2000), as predicted by the local chemokine paralysis model (Maekawa et al., 2014). These studies may help to elucidate that *P. gingivalis* plays an important role in the onset and development of periodontal damage and, consequently, in bone loss. Although the literature demonstrates that the action of *P. gingivalis* can be transient on the subversion of leukocytes (inhibited expression occurs only during the first days after oral inoculation of *P. gingivalis*), this subversive activity can delay the recruitment of neutrophils and allow initial biofilm formation in the relative absence of neutrophil defenses. Once a mature pathogenic biofilm develops that is able to resist neutrophil defenses, neutrophil recruitment can promote inflammation, thus contributing to the escalation of dysbiosis (de Molon et al., 2014; Zenobia & Hajishengallis, 2015).

In fact, not only does the inflammatory environment favor the development of a subset of inflammatory species derived from the key pathogen status of *P. gingivalis*, but compromised host immunity allows for uncontrolled bacterial growth, interrupting homeostasis and thus leading to selective inhibition of antimicrobial responses and promotion of destructive inflammation (Zenobia & Hajishengallis, 2015). These data suggest that, possibly, in an animal model, *P. gingivalis* has the capacity to create a favorable microenvironment for biofilm maturation, which may lead to the understanding that bone loss is a consequence for the groups where *P. gingivalis* gavage was executed. The identification of bone loss in the Sham group, a cohort typically subjected to procedures lacking the introduction of the experimental agent (*P. gingivalis* in this instance), may raise concerns. However, bone loss in the Sham group in periodontal disease studies can be attributed to various factors, encompassing stress associated with the gavage procedure (Lambert et al., 2019), disruptions in the oral microbiota (Abusleme et al., 2013), localized immune responses, potential contamination (Sanz et al., 2011), and strain‐specific variations in response (Gupta et al., 2019; Graves et al., 2012). The inclusion of Sham groups by researchers serves to control for these factors, ensuring that the observed bone loss is genuinely attributed to the experimental agent (*P. gingivalis*), rather than procedural effects.

When conducting research using an animal model of periodontal disease, many factors play a role in the degree and severity of the disease. Some of these factors include the species of animal, the method used to promote bone resorption (e.g., bacteria or ligature), specific bacteria used for infection, the study period following infection or ligature, and associated methods of disease assessment. The most commonly used measure to determine alveolar bone loss in mice is the distance from the CEJ to the ABC by means of magnification analysis or histomorphometry. The selection of the maxillary second molar is a pragmatic and scientifically well‐founded choice for probing *P. gingivalis* gavage‐induced bone loss in mice, offering advantages in terms of accessibility (Baker et al., 1994) anatomical similarity to the human dentition (Maresz et al., 2013), consistency between investigations (Hajishengallis et al., 2011), uniform experimental conditions and ethical considerations (Baker et al., 1994). These attributes make it an ideal candidate for investigating periodontal research in murine models. Our focus was on this specific bone area, assessing volumetric changes and identifying changes in bone mass during microCT analysis, following the protocol published by Shimabukuro et al. (2021). The hemimaxillae were scanned and a standard area in the interproximal region between the first and second molars, the second molar and the third molar was selected as the reference point in 15 coronal sections of the ECJ second molar as shown in our figure in question. Molon et al. (2014, 2016) also analyzed different models of periodontal disease (PD) induction, in which they inoculated mice with *P. gingivalis* at different intervals ranging from three to five times, and the authors did not find bone loss. Possibly the method of analysis may have been impaired to reach statistically significant differences. Although micro‐CT was used, only linear and area measurements were considered. Previous studies have already shown the need for adaptations to the model for measuring bone loss in mice inoculated with *P. gingivalis*. Wilensky et al. (2005) proposed a linear measurement in micro‐CT to detect changes in bone. The authors used the same model as Baker et al. (Baker, 2005) and found that the type of *P. gingivalis* strain could interfere with the results. However, the authors reported that there could be alterations in the alveolar bone that morphometric techniques probably could not capture. The measurement model used in the present study is capable of detecting volumetric changes in the alveolar bone crest, which may characterize greater sensitivity and guide future studies, in addition to adding bone microarchitecture details not reported by Wilensky et al. (2009). The interproximal bone crest appears to be an important point in detecting the onset of PD, where previous studies have reported changes in the alveolar bone crest as a predictor of periodontitis in humans (Rosier et al., 2018; Rosling et al., 1975; Zaki et al., 2015).

It is noteworthy that in this study, the C57BL/6 lineage disease profile was established after 2 weeks with a decrease in bone volume, increasing porosity and less organized trabecular pattern, which could be considered the peak of the disease. One of the hypotheses raised for difficulties in finding bone loss in gavage models is the time of administration of the pathogen. The studies conducted by Baker et al. in the 2000s used only three applications of *P. gingivalis* in 1 week which were evaluated 47 days later. Furthermore, another important point is that the authors did not administer antibiotics before PD induction, as demonstrated in Baker's studies. The previous administration of antibiotics seems to favor the reduction of the native microbiota and facilitate the insertion of the pathogen that can generate the dysbiotic picture. Furthermore, sensitization of the microbiota proves to be important when it is already established and has characteristics of resilience (Baker, Dixon, Evans, et al., 2000; Chen et al., 2004).

The differences in bone resorption observed between the 1‐week and 2–3‐week gavage groups with *P. gingivalis* could be attributed to the dynamics of periodontal disease progression, the host immune response, the virulence factors of the *P. gingivalis* strain used, and the intricate balance between bone resorption and formation (Hajishengallis, 2015). The dynamics of the host immune response can influence the progression of periodontal disease, leading to a process known as immune tolerance (Loos & Van Dyke, 2020). Longer study durations, host immune adaptation, and potential variations in virulence factors may explain why additional weeks of gavage did not significantly worsen bone resorption compared to the initial 1‐week period (Dudakovic et al., 2016). After 1 week of oral administration of *P. gingivalis* in mice, the observed bone loss is attributed to a multifaceted interplay of mechanisms. Firstly, *P. gingivalis*, a well‐known periodontal pathogen, triggers an inflammatory response in the oral cavity, involving the release of proinflammatory cytokines such as interleukin‐1 beta and tumor necrosis factor‐alpha. These cytokines activate osteoclasts, leading to initial bone loss (Graves et al., 2008). *P. gingivalis'*s virulence factors, including lipopolysaccharides and gingipains, directly stimulate bone resorption through various pathways, including activation of TLRs and degradation of host proteins (Hajishengallis et al., 2011; Lamont & Hajishengallis, 2015 *). P. gingivalis* disrupts periodontal tissues by invading and colonizing the gingival epithelium and subgingival crevices, prompting an inflammatory response and bone loss. *P. gingivalis*'s presence also alters the oral microbiota, promoting the proliferation of other pathogenic bacteria, further escalating inflammation and bone loss (Hajishengallis & Lamont, 2016; Hajishengallis et al., 2012). *P. gingivalis* infection activates the receptor activator of nuclear factor‐kappa B ligand (RANKL) pathway, increasing osteoclast activity and exacerbating bone resorption (Hajishengallis, 2014).

Beyond local effects, *P. gingivalis*‐induced periodontitis can have systemic repercussions, impacting distant organs and tissues, potentially contributing to bone loss (Olsen et al., 2016). The interconnected mechanisms highlight the complexity of *P. gingivalis*‐induced bone loss, emphasizing the significance of studying these processes in mouse models to understand periodontal disease pathogenesis and explore therapeutic interventions.

An important aspect of the present study is the use of the bacterial strain *P. gingivalis* W83, recognized for its high virulence profile (Dou et al., 2010; Sheets et al., 2005). Previous studies have shown that *P. gingivalis* W83 is capable of cleaving adhesion molecules such as N—and VE—cadherin and β1 integrin, which denotes increased tissue fragility and, through gingipain production, induces endothelial cell detachment and apoptosis (Hugoson & Laurell, 2000). These factors are associated with an increased inflammatory profile due to tissue degradation and increased pathogenicity, which may have impacted the results observed in bone tissue modifications.

Considering the chronicity of the induced periodontal lesion, it is important to understand that, unlike ligation models where there is rapid progression, the gavage model with *P. gingivalis* presents a slow progression, which further resembles the chronicity of a lesion in humans. Previous longitudinal studies in humans on the progression of periodontitis have shown that the rate of periodontal tissue destruction has a slow progression in most cases, and that advanced forms of the disease occur in some individuals and in a few dental sites, which denotes disease site specificity (Heitz‐Mayfield, 2005). Understanding risk factors can change outcomes when there are other conditions associated with PD (Ramseier et al., 2017).

Based on these results, the hypothesis that emerged is that systemic microbial challenge may alter host symbiosis and elicit the onset and/or progression of periodontal lesions. By altering the environment, infection may interfere with epigenetic factors, possibly causing susceptibility to expression (Palioto et al., 2019). The sum of these factors can guide future studies and help to elucidate the mechanism of pathogenesis, as well as new therapeutic approaches for periodontitis. Furthermore, future studies using real‐time assessment (in vivo micro‐CT) of disease activity and identification of local and systemic markers based on biomarkers, genotypes and phenotypes may improve the accuracy of measuring periodontal disease progression as well as possible changes in the microbiota for establishing the dysbiotic community.

To our knowledge, this study represents the first chronological description of the establishment of periodontal disease through systemic microbial challenge, specifically by *P. gingivalis* gavage. Within the limits of this research, it was deduced that 1 week of induction was adequate for the establishment of the disease in the BALB/c lineage, with both lineages showing well‐established disease after 2 weeks. Most parameters reached their peak 2 weeks after the microbial challenge, later stabilizing in the third week of induction. The rationale behind not evaluating the presence of *P. gingivalis* in gingival tissue is rooted in the study's main focus on investigating the consequences of gavage *P. gingivalis* administration on alveolar bone loss. The research question and study design were tailored to understand the mechanisms underlying bone loss and its impact on periodontal tissues, rather than directly addressing bacterial colonization. Therefore, the objectives of the study did not encompass the assessment of *P. gingivalis* colonization in gingival tissue, with an emphasis on unraveling the intricate facets of bone loss in the context of periodontal disease.

## AUTHOR CONTRIBUTIONS

Cristhiam de J. Hernandez Martinez contributed to the conceptualization, data curation, formal analysis, funding acquisition, investigation, methodology, and final approval of the version to be submitted. Pedro Felix Silva contributed to the analysis, software, validation, visualization, interpretation of data, and final approval of the version to be submitted. Sergio L. Salvador contributed to the methodology as well as writing the review and final approval of the version to be submitted. Michel Messora contributed to the design and analysis of the data, validation, visualization, roles/writing—original draft, and final approval of the version to be submitted. Daniela B‐. Palioto contributed to the conception, design and analysis of the data, supervision, validation, visualization, roles/writing—original draft, writing—review and editing, and final approval of the version to be submitted. All authors gave final approval and agreed to be accountable for all aspects of the work.

## CONFLICT OF INTEREST STATEMENT

The authors declare no conflict of interest.

## ETHICS STATEMENT

All experimental protocols were approved by the Ethical Committee on Animal Use of the Faculty of Dentistry of Ribeirão Preto‐University of São Paulo under the responsibility of PhD Daniela Bazan Palioto—which involves the production, maintenance‐, and/or use of animals belonging to the phylum Chordata, vertebrate subphylum (except man) for purposes of clinical or educational research—in accordance with the provisions of Law No. 11774 of October 8, 2008 of Decree No. 6899 of July 15, 2009 and the standards issued by the National Council for Control of Animal Experimentation (CONCEA) with protocol number CEUA/FORP 2018.1.644.58.6. All methods in this study were performed in accordance with relevant guidelines and regulations.

## Data Availability

The data supporting the findings presented in this manuscript are available upon request. Interested researchers may obtain access to the data by contacting the corresponding author: Daniela B. Palioto dpalioto@forp.usp.br (corresponding author). Cristhiam de J. Hernández Martínez seguidordexto@yahoo.es (first author). We believe that open and accessible data are essential for the advancement of scientific knowledge, and we are dedicated to facilitating the sharing of our research data with the research community
